# Drug resistance and extended-spectrum β-lactamase (ESBLs) - producing *Enterobacteriaceae, Acinetobacter* and *Pseudomonas* species from the views of one-health approach in Ethiopia: a systematic review and meta-analysis

**DOI:** 10.1186/s42522-023-00088-z

**Published:** 2023-09-11

**Authors:** Mengistu Abayneh, Ahmed Zeynudin, Rahel Tamrat, Mulualem Tadesse, Abraham Tamirat

**Affiliations:** 1 College of Medical and Health Science, Department of Medical Laboratory Sciences, Mizan-Tepi University, PO Box 260, Mizan-Aman, Ethiopia; 2 School of Medical Laboratory Sciences, Faculty of Health Sciences, Institute of Health, Jimma University, Jimma, Ethiopia; 3 Faculity of Public Health, Department of Health, Behavior and Society, Jimma University, Jimma, Ethiopia

**Keywords:** Drug resistance/ MDR, ESBL-production, Gram-negatives, Ethiopia

## Abstract

**Background:**

Although antimicrobial resistance (AMR) bacteria present a significant and ongoing public health challenge, its magnitude remains poorly understood, especially in many parts of the developing countries. Hence, this review was conducted to describe the current pooled prevalence of drug resistance, multidrug- resistance (MDR), and Extended-spectrum β-lactamase (ESBL)-producing *Enterobacteriaceae, Acinetobacter,* and *Pseudomonas* species in humans, the environment, and animals or food of animal origin in Ethiopia.

**Methods:**

PubMed, Google Scholar, and other sources were searched for relevant articles as per the preferred reporting items for systematic reviews and meta-analysis (PRISMA) guidelines. A critical appraisal for screening, eligibility, and inclusion in the meta-analysis was made based on the Joanna Briggs Institute’s (JBI) essential appraisal tools. The meta-analysis was done on Statistical Software Package (STATA) version 17.0.

**Results:**

A total of 33 research articles were included in this systematic review and meta-analysis. *Escherichia coli, Klebsiella* species, *Acinetobacter,* and *Pseudomonas* species were the most frequently reported bacteria from two or more sources. More than 50% of *Klebsiella* species and 25% to 89% of *Escherichia coli* from two or more sources were resistant to all analysed antibiotics, except carbapenems. Fifty-five percent (55%) to 84% of *Acinetobacter* species and 33% to 79% of *Pseudomonas* species from human and environmental sources were resistant to all analyzed antibiotics. Carbapenem resistance was common in *Acinetobacter* and *Pseudomonas* species (38% to 64%) but uncommon in *Enterobacteriaceae* (19% to 44%)*. Acinetobacter* species (92%), *Klebsiella* species (86%), and *Pseudomonas* species* (*79%) from human sources, and *Proteus* species (92%), and *Acinetobacter* species (83%), from environmental sources, were the common multidrug-resistant isolates. About 45% to 67% of *E. coli, Klebsiella*, *Acinetobacter,* and *Pseudomonas* species from human and environmental sources were ESBL producers.

**Conclusion:**

Our review report concluded that there was a significant pooled prevalence of drug resistance, MDR, and ESBL-producing *Enterobacteriaceae, Acinetobacter,* and *Pseudomonas* species from two or more sources. Hence, our finding underlines the need for the implementation of integrated intervention approaches to address the gaps in reducing the emergence and spread of antibiotic- resistant bacteria.

## Background

Antimicrobial resistance (AMR) remains a significant One- Health problem, affecting humans, animals, and the environment [1]. The infections caused by AMR bacteria are becoming more prevalent and can be difficult, and sometimes impossible to treat because the available drugs used to treat microbial infections have become less effective or ineffective. The AMR threat adds to the existing higher burden of bacterial infections, particularly in low- and middle-income settings in which there has been low access to adequate diagnostics, specifically at peripheral levels of the healthcare system. In addition to increased morbidity and mortality, resistant infections also add considerable costs to the healthcare system [1–3].

AMR gram-negative bacteria are the most frequently encountered bacterial isolates recovered from different clinical and non-clinical specimens [3]. The emergence of ESBL-producing and carbapenem-resistant gram-negative bacteria, particularly *Klebsiella pneumoniae*, *Escherichia coli*, *Acinetobacter baumannii,* and *Pseudomonas aeruginosa*, are a matter of national and international concern as they are an emerging cause of healthcare-associated infection (HAI) that pose a significant threat to human and animal health [4, 5]. The infections caused by these bacteria may not be treated with the available antibiotics due to high levels of resistance and are associated with poor treatment outcomes. Importantly, although there are existing knowledge gaps in understanding the transmission pathway of AMR bacteria, there are various routes for widespread transmission of resistance bacteria and genes between humans, animals and the surrounding environment [1, 6]. Resistant bacteria can spread across humans and animal communities, the food supply, healthcare facilities, and the environment, which increases the burden of resistance and antibiotic-resistant infections [6, 7].

Anyone of any age, in every country, can potentially be affected by the consequences of AMR. For instance, an estimated 4·95 million deaths were associated with bacterial AMR in 2019, and if not properly addressed, the numbers may be increase to 10 million per year by 2050 [8, 9]. The main factors exacerbating the issue of AMR in low-resource countries include limited access to quality antimicrobial drugs; antibiotics sold over the counter without prescriptions, or antibiotics used in feeding animals as prophylaxis or growth promoters. The issue of a lack of regulation and quality control of drugs, coupled with poor infection prevention and water, sanitation, and hygiene interventions, can accelerate the emergence and spread of drug-resistant microorganisms [10–13].

The ongoing public health threat of AMR bacteria was highlighted on the WHO list of critical-priorities for the need of new researches, discovery, and development of new antibiotics [14]. Ethiopia has also implemented the One Health approach to respond to the existing and emerging health security threats, including AMR [15]. However, poor integration among sectors, the institutionalization of One- Health as a good approach, limited research funds, and activities on One- Health are among the many challenges that need to be addressed. So far, no study has reported the current situation of AMR and ESBL-producing combinations in our country. Therefore, this systematic review and meta-analysis aimed to determine: I) the pooled prevalence of resistance to commonly prescribed broad-spectrum antibiotics; II) the pooled prevalence of MDR; and III) the pooled prevalence of ESBL-producing *Enterobacteriaceae, Acinetobacter,* and *Pseudomonas* species from humans, the environment, and animals, or food sources.

## Main text

### Data sources and search strategy

Objective and reproducible searches were made on PubMed and Google Scholar to find published articles related to our outcomes of interest. On PubMed, the following search string words were used: "drug resistance"[Mesh] OR "drug resistance, multiple, bacterial"[Mesh] OR "drug resistance, bacterial"[Mesh] OR "drug resistance, multiple"[Mesh] OR "drug resistance, microbial"[Mesh]) OR ("*Enterobacteriaceae*" [Mesh] OR "*Enterobacteriaceae* infections"[Mesh] OR "beta-lactamase, *Enterobacteriaceae*" [Supplementary concept]) OR ("*Acinetobacter* species"[Mesh] OR "*Acinetobacter baumannii*"[Mesh] OR "*Acinetobacter* infections"[Mesh] OR "beta-lactamase, *Acinetobacter baumannii*" [Supplementary concept] OR ("*Pseudomonas* species"[Mesh] OR "*Pseudomonas* infections"[Mesh] OR "*Pseudomonas aeruginosa*"[Mesh]) AND ("humans"[Mesh]) OR ("animals"[Mesh]) AND "human-animal interaction"[Mesh]) OR ("meat products"[Mesh]) OR ("poultry"[Mesh] OR "poultry products"[Mesh]) OR ("chicken"[Mesh]) OR ("cattle"[Mesh] OR "cattle diseases"[Mesh]) OR ("environment"[Mesh] OR "health facility environment"[Mesh]) AND ("Ethiopia"[Mesh]). The searching process was filtered by year of publication, from January 2014 to October 2022, and full-text research articles. Additionally, relevant studies were manually searched from the bibliographies of eligible studies and from other meta-analysis studies.

### Selection and eligibility criteria

The systematic and comprehensive literature review methods were used to identify, select, and critically appraise relevant research and to collect and analyze data from the studies that are included in the review. Those research articles conducted in Ethiopia and published in English as research articles in the years 2014 to 2022, and those articles focusing on the reports of antimicrobial-resistant *Enterobacteriaceae, Acinetobacter,* and *Pseudomonas* species in humans, animals, or food of animal origin, and those that provided details on the number of studied isolates, are used as criteria for eligibility for the review. On the other side, those articles that did not provide full information on the outcomes of interest, provided data on gram positives only, conducted molecular investigations of AMR molecular markers only, were not freely accessible as a full text, and those reviewed articles on AMR were excluded. In order to guarantee the quality of studies, two independent reviewers were assigned to select the articles throughout each stage of the review (i.e., screening, eligibility, and inclusion in meta-analysis).

### Article quality assessment

**Fig. 1:**
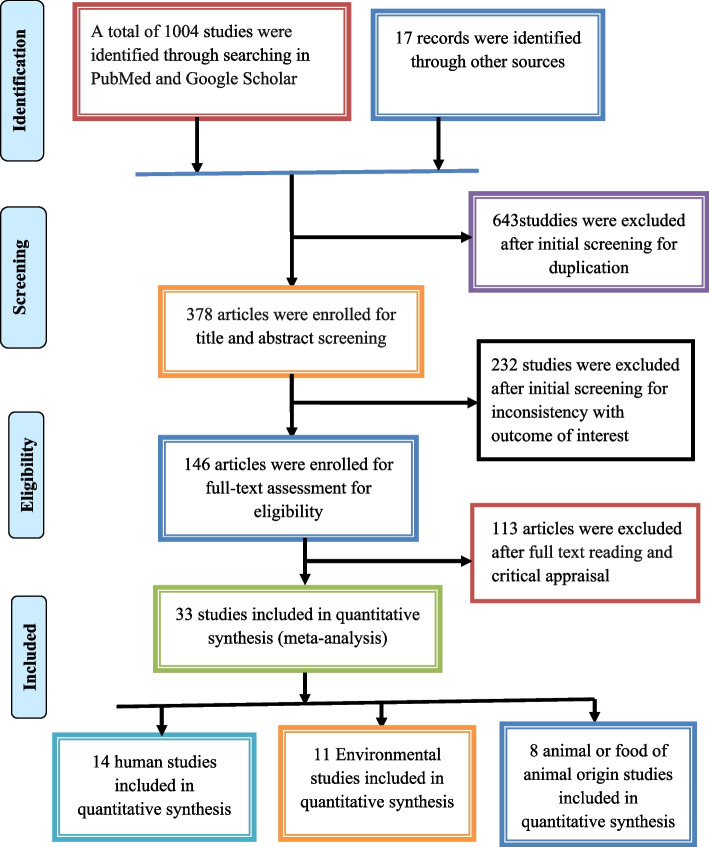
Flow diagram depicting the selection process of included articles

The article selection process was done based on the preferred reporting items for systematic reviews and meta-analysis (PRISMA) guidelines [16] (Fig. 1). The quality assessment and enrollment of each article were made by two independently critical appraisers based on the Joanna Briggs Institute (JBI) critical appraisal tools [17] and the Cochrane Handbook for Systematic Reviews [18]. The criteria for quality assessment include: whether the research question is clear and adequate to the study; whether the study design used is appropriate to the set research question; whether descriptions of the setting, including periods of recruitment, and the sampling method are appropriate for the set research question and design; and whether the collected data was properly managed and analyzed. In addition, a comprehensive search strategy was made in order to reduce the impact of publication bias on the results of the review.

### Data extraction

An Excel database was designed for the purpose of extracting data from the included studies. The first author, publication year, study region or area, study or data collection period, study design, study subjects, type of sample, type and numbers of selected gram-negative bacteria, the number of isolates tested for antimicrobial resistance, the number of isolates reported as MDR, and if reported, the numbers of ESBL producers were extracted. Additionally, the investigation method (phenotypic or genotypic) was extracted. The data extraction process was done independently and in duplicate using piloting forms to ensure double-checking.

### Data analysis

The total number of each bacterium species and the number of isolates tested for antimicrobial resistance from each source were extracted, and meta-analysis was done on STATA version 17. The pooled prevalence of AMR, MDR, and ESBL production for each bacterium was analyzed using the random-effects model. Cochran Q tests and the I^2^ statistic were used to analyze the heterogeneity of the studies, and significant variation was considered at *p*-values < 0.05 and I^2^ > 50% [19]. For the studies on the environment and food-producing animals, the meta-analysis was done if the outcome of interests was reported in at least three studies, whereas at least four studies were considered in the case of human sources. The pooled percentage for each reported resistant gram-negative species was then deduced from the total number of tested isolates. A categorical meta-analysis for each antibiotic resistance isolate was made based on their sources. Begg’s and Mazumdar rank correlation test was performed to assess the publication biases across the studies, and statistical significance was considered at a *p*-value < 0.05. Testing for publication bias and heterogeneity was carried out to check the extent of the variation in study outcomes between the included studies and whether the results of the studies were valid for systematic reviews and meta-analyses. Finally, the results were narrated in words and presented in figures and tables that were best suited for readers.

## Results

### General characteristics of the included studies

In this systematic review and meta-analysis, a total of 33 studies were included; of these, 14 were human studies, 11 were on environmental studies, and 8 were related to animals or foods of animal origin (Fig. 1). The included studies were published from 2014 to 2022, and 30 studies were done with a cross-sectional study design; two studies were retrospective and one was a cohort study. Based on the study area, half of human studies (50.0%) were from the Amhara region, 4 (36.4%) of the environmental studies were from southern Ethiopia, and 3 (37.5%) studies on animals or foods of animal origin were from Addis Ababa (Table 1).

**Table 1:** General characteristics of included studies (2014- 2022)

**Study region**	**Study Year**	**Publication Year**	**Study Design**	**Sample size**	**Sources and types of samples**	**Method**	**Positive samples**	**References**
**Human related studies**								
Amhara	April 1 to July, 2018	2020	CS	238	Multiple clinical specimens from patients with nosocomial infections	Phenotypic	20	Motbainor H, et al., 2018 [20]
Amhara	March to June 2019	2020	CS	153	Sputum samples from patients with respiratory conditions	Phenotypic and genotypic	78	Abda EM. et al*.* 2020 [21]
Amhara	Dec. 2017- April 2018	2021	CS	833	Multiple clinical samples from different infection sites	Phenotypic	141	Moges F. et al*.* 2021 [22]
Amhara	2011 to 2014	2017	RCS	575	Multiple clinical samples from different infection sites	Phenotypic	280	Mulu W. et al. 2017 [23]
Amhara	January to May 2017	2020	CS	166	Blood specimen from puerperal sepsis post-partum/aborted women	Phenotypic	56	Admas A. et al*.* 2020 [24]
Amhara	Feb. to April, 2020	2021	CS	254	Multiple clinical specimens from patients with nosocomial infections	Phenotypic	33	Mekonnen H, et al. 2021 [25]
Amhara	Feb.–Aug. 2021	2022	CS	423	Multiple clinical specimens from patients with nosocomial infections	Phenotypic	75	Tilahun M. et al., 2022 [26]
Addis Ababa	March and Dec. 2017	2021	Cohort	119	Blood specimens from newborns with gram-negative sepsis	Phenotypic	119	Solomon S, et al. 2021 [27]
Addis Ababa	June, 2019 to May, 2020	2021	CS	1,337	Multiple clinical samples from different infection sites	Phenotypic	429	Abdeta A, et al*.* 2021 [28]
Addis Ababa	Oct. 2016 to Sep-2017	2019	CS	996	Multiple clinical samples from different infection sites	Phenotypic	135	Bitew A, 2019 [29]
Addis Ababa	Sep. 2018 to Jan. 2019	2022	CS	2397	Blood samples from patients with blood stream infections	Phenotypic and genotypic	597	Seman A. et al*.* 2022 [30]
Oromia	May to Sep., 2016	2018	CS	197	Multiple clinical specimens from patients with nosocomial infections	Phenotypic	118	Gashaw M. et al*.* 2018 [31]
Oromia	April 2016 to June 2018	2022	CS	684	Multiple clinical samples from different infection sites	Phenotypic and genotypic	65	Tufa BT., et al*.* 2022 [32]
South Ethiopia	Five-year (2016–2020)	2022	RCS	581	Multiple clinical samples from different infection sites	Phenotypic	237	Ageru TA. et al. 2022 [33]
**Environmental studies**								
Amhara	May 2016-Aug 2016	2021	CS	110	Leafy vegetable samples	Phenotypic and genotypic	23	Cherinet Y. et al*.*2021 [34]
Amhara	January-June 2012	2014	CS	60	Hospital environment waste water samples	Phenotypic	51	Moges F. et al*.* 2014 [35]
Amhara	Dec. 2020 to Mar. 2021	2021	CS	384	Swabs of hospital contact surfaces, leftover drugs and 80% ethanol	Phenotypic	102	Firesbhat A, et al*.* 2021 [36]
Addis Ababa	Jan. to April 2019	2021	CS	572	Swab samples from HCW mobile phone	Phenotypic	454	Araya S. et al*.* 2021 [37]
Addis Ababa	June to Sep.2018	2020	CS	164	Hospital environment swab samples	Phenotypic	141	Sebre S. et al*.* 2020 [38]
Addis Ababa	Feb. to April, 2017	2018	CS	94	River water samples	Phenotypic	90	Belachew T. et al*.* 2018 [39]
South Ethiopia	Feb. to April,2021	2022	CS	120	Hospital Indoor air samples	Phenotypic	120	Kayta G, et al*.* 2022 [40]
South Ethiopia	May to June, 2018	2021	CS	99	Swab samples from hospital contact surfaces	Phenotypic	71	Birru M, et al*.* 2018 [41]
South Ethiopia	Nov 2014 to Feb,2015	2016	CS	120	Hospital Indoor air samples	Phenotypic	120	Hailemariam M, et al*.* 2016 [42]
South Ethiopia	Dec. to April,2015	2017	CS	216	Hospital Indoor air samples	Phenotypic	67	Solomon FB. et al*.* 2017 [43]
Tigray	Oct. 2016 to June 2017	2019	CS	130	Swab samples from hospital contact surfaces	Phenotypic	115	Darge A, et al*.* 2019 [44]
**Studies on animal or food of animal origin**								
Oromia	April to June, 2018	2021	CS	140	Fresh chicken dropping from poultry farms	Phenotypic	61	Bushen A, et al*.* 2021 [45]
Amhara	Feb. to Mar., 2012	2014	CS	44	Poultry wastes from poultry farms	Phenotypic	52	Eyasu A. et al*.* 2014 [46]
South Ethiopia	Sep. to Dec. 2020	2022	CS	556	Raw cattle meat and meat cutting equipment at butcher houses	Phenotypic	36	Worku W. et al*.* 2022 [47]
Addis Ababa	Aug. 2019 to July 2021	2022 Unpublished	CS	642	Cow’s raw milk from dairy farms and milk selling points, Meat/carcass swab of cattle, sheep, goat, and chicken from butcher houses, supermarkets and abattoirs and animal feed samples from feed manufacturing plants	Phenotypic	185	Tefera B, et al*.* 2022 [48]
Oromia	Dec., 2013 to May, 2014,	2020	CS	384	Samples from caecal contents of chicken	Phenotypic	56	Asfaw Ali D. et al.2020 [49]
Amhara	Feb. 2014 and Dec. 2015	2016	CS	384	Egg sandwich, minced and raw meat, burger patties, cottage cheese, cream cake, and beef pizza from restaurants, cafeterias, and pastry and retail shops Raw egg and pasteurized and raw milk from supermarkets and retail shops	Phenotypic	21	Ejo M, et al*.*2016 [50]
Addis Ababa	Dec. 2014 to April 2015	2016	CS	280	Lung and liver swab samples from bovines and ovines slaughtered at abattoir house	Phenotypic	13	Kebede A et al*.* 2016 [51]
Addis Ababa	Aug. 2011 to April 2012	2014	CS	384	Meat samples of animals from abattoir and retailers shops	Phenotypic	39	Bekele T et al*.* 2014 [52]

Out of the 14 included studies on humans, 10 studies involved various clinical samples for the detection of drug-resistant bacteria from patients with multiple infections. Bloodstream infections (BSIs), urinary tract infections, nosocomial infections, and other conditions are commonly considered medical conditions from which drug-resistant bacteria isolates were reported. In studies involving animals or foods of animal origin, raw milk, meat or carcass swabs, animal feeds, and chicken droppings and caecum were the most frequently considered specimens in the detection of drug-resistant isolates. Swabs from hospital contact surfaces and mobile phones, indoor air, and waste/river water are the sources of samples for environmental studies. The detailed characteristics of the studies are presented below in Table 1.

In this review, Begg’s and Mazumdar rank correlation test showed that no significant effect of publication bias was observed among the included studies (*p*-value > 0.05). However, the variation in the study methodology, setups, study periods, and study populations could have an effect on the heterogeneity among the included studies.

### The frequency of selected bacterial isolates

**Fig. 2:**
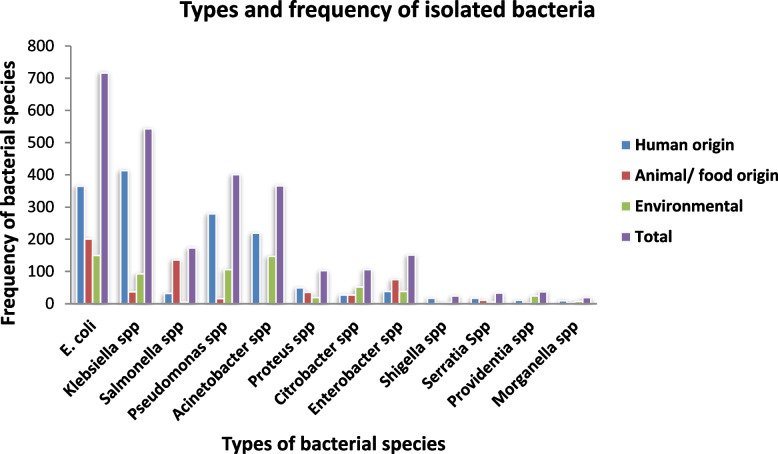
Type and frequency of bacteria isolated from humans, environment and from animals or food of animal origin

In this review, a total of 12 species of gram-negative bacteria were extracted; however, the meta-analysis was computed for eight gram-negative bacteria from studies in humans, the environment, and animals, or food of animal origin. *Escherichia coli* (*n* = 716), *Klebsiella* species (*n* = 543), *Pseudomonas* species (*n* = 401), and *Acinetobacter* species (*n* = 366) were the most frequently reported species from two or more sources (Fig. 2).

### The pooled prevalence of AMR for selected bacterial isolates

The pooled prevalence of AMR for each bacterium-antibiotic combination in each source was estimated using a random effect model. Accordingly, from isolates of humans, *E. coli* was reported to have a high proportion of pooled resistance to ampicillin (0.89; 95% CI: 0.81, 0.94), co-trimoxazole (0.83; 95% CI: 0.72, 0.91), ceftriaxone (0.79; 95% CI: 0.65, 0.88), ciprofloxacillin (0.77; 95% CI: 0.63, 0.87), and gentamycin (0.73; 95% CI: 0.56, 0.85). As *E. coli, Klebsiella* spp. showed a higher proportion of resistance to co-trimoxazole (0.82; 95% CI: 0.71, 0.90), ceftriaxone (0.80; 95% CI: 0.67, 0.88), ciprofloxacillin (0.73; 95% CI: 0.58, 0.85), and gentamycin (0.78; 95% CI: 0.65, 0.87), but relatively lower rates of resistance were observed to meropenem (0.38; 95% CI: 0.14, 0.70). However, a proportion of 0.64 (95% CI: 0.48, 0.78) *Acinetobacter* species and 0.55 (95% CI: 0.33, 0.74) *Pseudomonas* species was resistant to meropenem (Table 2).

**Table 2:** Estimated AMR gram-negative bacteria isolated from humans, animals/food, and the environment

**Bacterial type**	**Sources of isolates**	**# of isolates**	**Types of antibiotics and estimated resistance (95% CI)**									
			**AMP**	**AMC**	**CRO**	**CTX**	**CTZ**	**CEF**	**CIP**	**CN**	**SXT**	**MRP**
*E. coli*	Human	312	0.89 (0.81, 0.94)	0.77 (0.65, 0.86)	0.79 (0.65, 0.88)	0.74 (0.58, 0.85)	0.79 (0.65, 0.88)	0.76 (0.61, 0.87)	0.77 (0.63, 0.87)	0.73 (0.56, 0.85)	0.83 (0.72, 0.91)	0.19 (0.03, 0.67)
	Animal/ Food	201	0.79 (0.68, 0.87)	0.50 (0.31, 0.69)	0.25 (0.07, 0.59)	0.31 (0.11, 0.62)	0.43 (0.22, 0.67)	ND	0.29 (0.10, 0.61)	0.27 (0.09, 0.60)	0.51 (0.31, 0.71)	ND
	Environment	93	0.78 (0.67, 0.85)	0.61 (0.47, 0.73)	0.63 (0.50, 0.75)	0.41(0.25, 0.60)	0.47 (0.31, 0.63)	0.57 (0.43, 0.70)	0.54 (0.40, 0.69)	0.48 (0.32, 0.64)	0.61(0.48, 0.73)	0.26 (0.11, 0.50)
*Klebsiella* spp	Human	226	0.71 (0.55, 0.84)	0.80 (0.68, 0.88)	0.80 (0.67, 0.88)	0.74 (0.59, 0.85)	0.80 (0.67, 0.88)	0.79 (0.66, 0.88)	0.73 (0.58, 0.85)	0.78 (0.65, 0.87)	0.82 (0.71, 0.90)	0.38 (0.14, 0.70)
	Animal/ Food	-	ND	ND	ND	ND	ND	ND	ND	ND	ND	ND
	Environment	150	0.82 (0.72, 0.89)	0.68 (0.54, 0.79)	0.60 (0.44, 0.74)	0.52 (0.34, 0.69)	0.44 (0.25, 0.65)	0.52 (0.34, 0.69)	0.59 (0.43, 0.74)	0.58 (0.41, 0.73)	0.70 (0.57, 0.81)	0.44 (0.25, 0.65)
*Pseudomonas* spp	Human	257	0.79 (0.67, 0.87)	0.70 (0.54, 0.82)	0.75 (0.61, 0.85)	0.34 (0.12, 0.66)	0.71 (0.55, 0.82)	0.50 (0.28, 0.72)	0.71 (0.56, 0.83)	0.69 (0.52, 0.81)	0.73 (0.59, 0.84)	0.55 (0.33, 0.74)
	Animal/ Food		ND	ND	ND	ND	ND	ND	ND	ND	ND	ND
	Environment	106	0.57 (0.42, 0.71)	0.33 (0.16, 0.55)	0.59 (0.44, 0.72)	0.61 (0.46, 0.73)	0.54 (0.39, 0.69)	0.63 (0.49, 0.75)	0.66 (0.53, 0.77)	0.46 (0.29, 0.63)	0.64 (0.50, 0.75)	0.38 (0.21, 0.58)
*Acinetobacter* spp	Human	199	0.68 (0.52, 0.80)	0.66 (0.50, 0.79)	0.78 (0.67, 0.87)	0.79 (0.67, 0.87)	0.82 (0.72, 0.89)	0.73 (0.60, 0.83)	0.78 (0.66, 0.86)	0.79 (0.67, 0.87)	0.82 (0.72, 0.89)	0.64 (0.48, 0.78)
	Animal/ Food	-	ND	ND	ND	ND	ND	ND	ND	ND	ND	ND
	Environment	133	0.70 (0.57, 0.80)	0.62 (0.46, 0.75)	0.81 (0.71, 0.88)	0.74 (0.62, 0.83)	0.77 (0.66, 0.85)	0.82 (0.72, 0.89)	0.74 (0.63, 0.83)	0.78 (0.67, 0.86)	0.84 (0.75, 0.90)	0.55 (0.38, 0.71)
*Salmonella* spp	Human	-	ND	ND	ND	ND	ND	ND	ND	ND	ND	ND
	Animal/ Food	136	0.66 (0.52, 0.78)	0.61 (0.45, 0.74)	0.24 (0.08, 0.53)	ND	ND	ND	ND	0.39 (0.21, 0.61)	0.63 (0.48, 0.76)	ND
	Environment	-	ND	ND	ND	ND	ND	ND	ND	ND	ND	ND
*Proteus* spp	Human	44	0.76 (0.66, 0.83)	0.54 (0.42, 0.66)	0.61 (0.49, 0.71)	0.32 (0.20, 0.48)	0.59 (0.47, 0.80)	0.62 (0.51, 0.72)	0.56 (0.44, 0.67)	0.46 (0.33, 0.59)	0.76 (0.66, 0.83)	ND
	Animal/ Food	-	ND	ND	ND	ND	ND	ND	ND	ND	ND	ND
	Environment	17	0.28 (0.18, 0.39)	0.48 (0.38, 0.58)	0.15 (0.08, 0.37)	ND	ND	0.08 (0.03, 0.18)	0.22 (0.13, 0.34)	0.33 (0.23, 0.45)	0.58 (0.48, 0.68)	ND
*Citrobacter* spp	Human	23	1.00 (0.96, 1.00)	0.68 (0.59, 0.77)	0.61 (0.51, 0.70)	ND	0.47 (0.36, 0.58)	0.51 (0.40, 0.61)	0.40 (0.29, 0.62)	0.47 (0.36, 0.58)	0.61 (0.51, 0.70)	ND
	Animal/ Food	-	ND	ND	ND	ND	ND	ND	ND	ND	ND	ND
	Environment	54	0.67 (0.56, 0.77)	0.51 (0.38, 0.64)	0.14 (0.05, 0.35)	ND	ND	ND	0.36 (0.22, 0.52)	0.33 (0.20, 0.50)	0.44 (0.30, 0.58)	ND
*Enterobacter* spp	Human	33	0.61(0.50, 0.71)	0.50 (0.39, 0.62)	0.68 (0.57, 0.76)	ND	0.63 (0.53, 0.73)	0.57 (0.46, 0.67)	0.38 (0.26, 0.51)	0.50 (0.39, 0.62)	0.61 (0.51, 0.71)	ND
	Animal/ Food	-	ND	ND	ND	ND	ND	ND	ND	ND	ND	ND
	Environment	38	0.59 (0.48, 0.69)	0.24 (0.13, 0.40)	0.20 (0.09, 0.37)	0.04 (0.01, 0.22)	0.04 (0.01, 0.22)	0.34 (0.21, 0.48)	0.27 (0.16, 0.43)	0.47 (0.34, 0.59)	0.56 (0.44, 0.67)	0.24 (0.13, 0.40)

*NBAMP* Ampicillin, *AMC* Amoxicillin-clavunilic acid, *CRO* Ceftriaxone, *CAZ* Ceftazidime, *CTX* Cefotaxime, *CEF* Cefepime, *CIP* Ciprofloxacin, *CN* Gentamycin, *SXT* Trimethoprim- sulphamethoxazole, *MRP* Meropenem*,* and Pooled prevalence of AMR was not calculated for *Shigella spp*. (*n* = 24), *Serratia* spp. (*n* = 33), *Providentia spp* (*n* = 37) and *Morganella spp* (*n* = 19) because, the total number of isolates tested for antimicrobial resistance from a two or more sources was < 50. “ND” was used to indicate susceptibility testing was not performed to calculate pooled prevalence of AMR

Among the isolates from the environmental sources, *Klebsiella* species accounted for the highest proportion of pooled resistance to ampicillin (0.82; 95% CI: 0.72, 0.89), amoxicillin-clavunilic acid (0.68; 95% CI: 0.54, 0.79), ceftriaxone (0.60; 95% CI: 0.44, 0.74), and co-trimoxazole (0.70; 95% CI: 0.57, 0.81). *E. coli* was also reported to have a high rate of pooled resistance to ampicillin (0.78; 95% CI: 0.67, 0.85), ceftriaxone (0.63; 95% CI: 0.50, 0.75), and co-trimoxazole (0.61; 95% CI: 0.48, 0.73). More than 70% of *Acinetobacter* species were resistant to most tested antibiotics, specifically ceftriaxone (0.81; 95% CI: 0.71, 0.88), co-trimoxazole (0.84; 95% CI: 0.75, 0.90), gentamycin (0.78; 95% CI: 0.67, 0.86), and ciprofloxacillin (0.74; 95% CI: 0.63, 0.83). A high proportion of resistance was also reported by *Pseudomonas* species to ceftriaxone (0.59; 95% CI: 0.44, 0.72), ciprofloxacillin (0.66; 95% CI: 0.53, 0.77), and co-trimoxazole (0.64; 95% CI: 0.50, 0.75). Resistance to meropenem was observed in 0.55 (95% CI: 0.38, 0.71) of *Acinetobacter* species, in 0.44 (95% CI: 0.25, 0.65) of *Klebsiella* spp., and in 0.38 (95% CI: 0.21, 0.58) of *Pseudomonas* species (Table 2).

Among isolates from animals or food of animal origin, the highest proportions of resistance to ampicillin (0.79; 95% CI: 0.68, 0.87), amoxicillin-clavunilic acid (0.50; 95% CI: 0.31, 0.69), and co-trimoxazole (0.51; 95% CI: 0.31, 0.71) were reported in *E. coli*. *Salmonella* species also showed the highest proportion of resistance to ampicillin (0.66; 95% CI: 0.52, 0.78), amoxicillin-clavunilic acid (0.61; 95% CI: 0.45, 0.74), and co-trimoxazole (0.63; 95% CI: 0.48, 0.76) (Table 2).

### The pooled proportion of MDR bacterial isolates

In this review, the pooled prevalence of MDR for each bacterium was computed from the forest plots and was only calculated when the total number of isolates tested for multidrug resistance from the sectors was ≥ 50. Among human isolates, *Acinetobacter* species showed the highest pooled proportion of MDR (0.92; 95% CI: 0.75, 1.00), followed by *Klebsiella* species (0.86; 95% CI: 0.64, 0.98), and *Pseudomonas* species (0.79; 95% CI: 0.61, 0.93). Among the isolates from environmental studies, the highest proportion of MDR was found in *Proteus* species (0.94; 95% CI: 0.89, 0.97), *Acinetobacter* species (0.83; 95% CI: 0.45, 1.00), and *Klebsiella* species (0.70; 95% CI: 0.32, 0.98). In the case of isolates from animals or food of animal origin, *E. coli* and *Salmonella* species were reported with a pooled MDR of 0.36 (95% CI: 0.24, 0.50) and 0.29 (95% CI: 0.12, 0.42), respectively (Table 3).

**Table 3:** Estimated rate of MDR in gram-negative bacteria from humans, animals/food, and the environment

Type of bacteria	Sources of isolates and estimated multidrug- resistance (95% CI)			Overall pooled MDR: ES (95%CI), I^2^ = % *p* = value	Heterogeneity of the studies
	**Humans**	**Animals/Food**	**Environment**		
*E. coli*	0.43 (0.23, 0.63)	0. 36 (0.24, 0.50)	0.42 (0.21, 0.65)	0.41 (0.30, 0.53), I^2^ = 93.17% *p* = 0.000	No, *p* = 0.573
*Klebsiella* spp	0.86 (0.64, 0.98)	―	0.70(0.32, 0.98)	0.80(0.61, 0.96), I^2^ = 97.38% *p* = 0.000	No, *p* = 0.409
*Salmonella* spp	―	0.29 (0.12, 0.42)	―	I^2^ = 89.78% *p* = 0.000	―
*Pseudomonas* spp.	0.79 (0.61, 0.93)	―	0.54 (0.47, 0.62)	0.74 (0.57, 0.88), I^2^ = 96.79% *p* = 0.000	Yes, *p* = 0.015
*Acinetobacter* spp.	0.92 (0.75, 1.00)	―	0.83 (0.45, 1.00)	0.89 (0.74, 0.98), I^2^ = 97.01% *p* = 0.000	No, *p* = 0.573
*Proteus* spp.	0.33 (0.08, 0.64)	―	0.94 (0.89, 0.97)	0.48 (0.13, 0.83), I^2^ = 98.60% *p* = 0.000	Yes, *p* = 0.000
*Citrobacter* spp.	―	―	0.39 (0.05, 0.81)	I^2^ = 98.84%, *p* = 0.000	-
*Enterobacter* spp.	0.41 (0.34, 0.49)	―	0.55(0.02, 1.00)	0.47(0.11, 0.86), I^2^ = 98.85%, *p* = 0.000	No, *p* = 0.692

### The pooled prevalence of ESBL- production

In this review, the rate of ESBL production was also computed from the forest plots for each bacterium. Among human isolates, the highest proportion of ESBL production was recorded by *Pseudomonas* species (0.67; 95% CI: 0.55, 0.77), followed by *Klebsiella* species and *E. coli* each was (0.59; 95% CI: 0.46, 0.70) and *Acinetobacter* species (0.56; 95% CI: 0.44, 0.68). Among the isolates from environmental studies, the highest proportion of ESBL production was found in *Acinetobacter* species (0.66; 95% CI: 0.54, 0.76), *Klebsiella* species (0.62; 95% CI: 0.51, 0.72), and *Pseudomonas* species (0.48; 95% CI: 0.36, 0.61) (Table 4).

**Table 4:** Estimated ESBL-producers among gram-negative bacteria isolated from humans and the environment

Type of bacteria	Sources of isolates and estimated ESBL-production (95%CI)	
	**Humans**	**Environment**
*E. coli*	0.59 (0.46, 0.70)	0.45 (0.34, 0.56)
*Klebsiella* spp	0.59 (0.46, 0.70)	0.62 (0.51, 0.72)
*Pseudomonas* spp	0.67 (0.55, 0.77)	0.48 (0.36, 0.61)
*Acinetobacter* spp	0.56 (0.44, 0.68)	0.66 (0.54, 0.76)
*Proteus spp*	0.40 (0.31, 0.51)	0.47 (0.38, 0.56)
*Citrobacter spp*	0.28 (0.19, 0.39)	0.26 (0.17, 0.37)
*Enterobacter spp*	0.40 (0.31, 0.51)	0.10 (0.05, 0.21)
**Random pooled prevalence: (95%CI), I**^**2**^** = % *p* = value**	0.50 (0.39, 0.60), I^2^ = 82.97% *p* = 0.000	0.43 (0.29, 0.57), I^2^ = 91.21% *p* = 0.000

## Discussion

This systematic review and meta-analysis was conducted to estimate drug- and multidrug-resistant bacteria from one-health perspective in Ethiopia. It also determined the prevalence of ESBL-producing gram-negative bacteria in human and environmental isolates. From human sources, more than 60% resistance was reported to commonly prescribed β-lactam antibiotics, ciprofloxacillin, gentamycin, and co-trimoxazole. In addition, the highest rates of MDR were found in *Acinetobacter* spp. (92%), followed by *Klebsiella* species (86%), and *Pseudomonas* species (79%). With some exceptions, almost consistent findings were reported in a review of findings in Ethiopia [53, 54], and in Cameroon [55], and East Africa [56]. Hence, this review suggests that, as infections caused by antibiotic- resistant bacteria are becoming more prevalent, serious concerns should be given to the use and choice of antibiotics for effective management of infections in Ethiopia.

Gram-negative bacteria use several mechanisms to develop resistance to antimicrobials. Mutations and recombination of genomic materials allow these bacteria to disseminate genes encoding for antimicrobial resistance within and across species [57]. Actions in the human and animal healthcare sectors are all considered to be contributing to the development of pathogen resistance to current available antimicrobials [57–60]. Frequent use of antibiotics may create favorable conditions for selective pressure, which leads to the further development of resistance. For instance, the production of β-lactamase that hydrolyzes the β-lactam ring is the most common resistance mechanism for these bacteria against β-lactam antibiotics. Gram-negative bacteria that produce ESBLs carry plasmid-encoded enzymes that can hydrolyze and confer resistance to a variety of β-lactam antibiotics, as well as fluoroquinolones, aminoglycosides, and trimethoprim-sulfamethoxazole [57, 61, 62].

In this review, above 50% of *E. coli*, *Klebsiella*, *Pseudomonas*, and *Acinetobacter* species from human sources were ESBL producers. The presence of bacteria in human and animal bodies as carriers may result in frequent exposure to antimicrobials used for treatment and prophylactic purposes [57, 59, 60, 62, 63]. There is no question that the widespread use, overuse, and misuse of antimicrobials have been associated with the explosion of antimicrobial resistance. A study confirmed that those who had exposure to third-generation cephalosporins, carbapenems, and fluoroquinolones had three-to-four times greater risk for infections with extended-spectrum β-lactamase-producing bacteria [64]. Therefore, updated and effective measures, such as antimicrobial stewardship which promotes the careful and responsible use of antimicrobials and prevents antimicrobial overuse and misuse in hospital and community settings, and infection prevention, are the most effective ways to reduce the spread and development of antimicrobial resistance and to protect patients from harms caused by unnecessary antibiotic use.

Antimicrobial susceptibility testing appeared to be inconsistent and low in animal, food, and environmental sources of isolates compared with humans. From environmental sources, *E. coli, Klebsiella* spp., and *Acinetobacter* spp. were recorded with more than 60% rates of AMR to ampicillin, amoxicillin-clavulanic acid, ceftriaxone, and co-trimoxazole. The rate of MDR was above 50% for five bacterial species. Mutation of bacterial genomes by different mechanisms, such as frequent antibiotic use or misuse in long-care facilities, may provide a selective advantage to the emergence of resistant variants [57, 59, 61]. For instance, in this review, 10 to 66% of the ESBL- production rate was found in environmental isolates, with the highest rates found in *Acinetobacter* (66%) and *Klebsiella* spp. (62%). Most of the included environmental studies were from hospital settings, specifically hospital surfaces, indoor air, and wastewater, suggesting a need for control of resistant gram-negative infections through a comprehensive approach, including detection and identification of resistant organisms and implementation of effective infection-control and prevention strategies in healthcare settings.

In isolates from animals or food of animal origin, the analysis for drug resistance was done only for *E. coli* and *Salmonella* species. Accordingly, greater than 50% of *E. coli* and *Salmonella* species were resistant to ampicillin, AMC, and co-trimoxazole, and the rate of MDR was 36% and 29%, respectively. A higher pooled estimate of antibiotic resistance (86%) and multidrug resistance (73%) was also reported in a review study in Africa [65]. Surface contamination with fecal matter, animal excreta, and water or soil sources may allow the transmission of drug-resistant bacterial populations to raw meat and carcasses, which could be transmitted to humans through consumption of animal products [66–69]. Additionally, the frequent contact between humans, dairy cattle, and poultry may also be a good opportunity for the bidirectional transmission of AMR bacteria such as *E. coli* [60, 69, 70]. Hence, the frequent contact with dairy cattle and poultry products as well as the habitual consumption of raw meat and milk may be contributing factors in the acquisition of resistance bacteria.

In general, in this review study, the prevalence of AMR, MDR, and ESBL-producing bacteria was higher in isolates from human samples as compared to other environmental and animal samples. However, some isolates from hospital environments showed comparable rates of AMR, MDR, and ESBL production. This may be indicated by the frequent exposures of humans to most antibiotics and the healthcare sectors, which can be contributing factors to the development of resistance and the possible transmission of antimicrobial- resistant bacteria from humans to the hospital environment and vice versa. Therefore, implementation of the integrated approaches, such as best regulation of the use of antibiotics, effective infection prevention, improving food safety, and preventing zoonotic disease infections, are important measures for the prevention and control of these complex AMR development and transmission cycles.

## Conclusion

This review report consists of the most recent situation of AMR with commonly prescribed antibiotics from a one-health perspective in Ethiopia. The review indicated that the high pooled prevalence of antibiotic resistance, MDR, and ESBL-production was in *Enterobacteriaceae*, *Acinetobacter,* and *Pseudomonas* species isolated from humans, the environment, and animals or food of animal origin. Therefore, to address the gaps related to measures taken to reduce the emergence and spread of AMR bacteria in humans, animals, and the environment, it is time to implement a harmonized and multidisciplinary one-health approach.

## Data Availability

All the data generated and analyzed during this review are included in this published article in the form of the main tables, but on reasonable request, details of our analysis are available from the corresponding author.

## Abbreviations

AMR: Antimicrobial resistance
ESBL: Extended-spectrum β-lactamase
MDR: Multidrug-resistance
PRISMA: Preferred reporting items for systematic reviews and meta-analysis
JBI: Joanna Briggs Institute
STATA: Statistical Software Package
